# The effect on participation rates of including focused spirometry information in a health check invitation: a cluster-randomised trial in Denmark

**DOI:** 10.1186/s12889-019-7531-5

**Published:** 2019-08-28

**Authors:** Lene Maria Ørts, Anders Løkke, Anne-Louise Bjerregaard, Helle Terkildsen Maindal, Kasper Norman, Bodil Hammer Bech, Annelli Sandbæk

**Affiliations:** 1 Department of Public Health, Aarhus University, Bartholins Allé 2, 8000 Aarhus, Denmark; 2 Department of Respiratory Diseases, Vejle Hospital, Vejle, Denmark

**Keywords:** Preventive health services, Patient participation, Lung diseases

## Abstract

**Background:**

Early detection of lung disease may help reduce disease development. Detection through preventive health checks may be beneficial. Nevertheless, the knowledge is sparse on how to enhance the participation rate in health checks among citizens at risk of developing lung disease. This study investigates if focused information on spirometry can increase the participation rate in a general health check.

**Methods:**

We conducted an open-label, household cluster-randomised trial with a two-group parallel design including 4407 citizens aged 30–49 years in Denmark and an average cluster size of 1.55 citizens per household. The control group (*n* = 2213) received a standard invitation describing the content of the general health check and containing practical information. The intervention group (*n* = 2194) received an extended invitation highlighting the benefits of early detection and prevention of lung disease. The primary outcome was difference in participation rate between the two groups. The secondary outcome was the proportion of participants at risk of lung disease in both groups. Risk profile was defined as current smoking or self-reported lung symptoms. The inclusion period was 25 November 2015–3 February 2017.

**Results:**

No major difference in participation rate was seen between the intervention group (53.4%) and the control group (52.0%). Participants had statistically significantly higher education level compared to non-participants. A total of 24.2% of the participants were at risk of developing lung disease, but no difference was found between the intervention group and the control group.

**Conclusion:**

This study revealed no effect on participation rate of including focused spirometry information in the health check invitation.

**Trial registration:**

ClinicalTrials.gov: NCT02615769. Registered on 25 November 2015. The trial protocol has been published.

**Electronic supplementary material:**

The online version of this article (10.1186/s12889-019-7531-5) contains supplementary material, which is available to authorized users.

## Background

Spirometry measurement is the gold standard for detection of poor lung function [1, 2]. Early detection and targeted treatment of individuals with poor lung function may reduce the risk of developing chronic lung disease [1, 2]. Treatment consists of pharmacological therapy and non-pharmacological treatment, such as smoking cessation, physical activity, pulmonary rehabilitation, and influenza and pneumococcal vaccinations [2, 3].

The Danish Health Authority recommends spirometry to all individuals above age 35 with at least one respiratory symptom (dyspnoea, cough, wheeze or sputum production) or risk factor (current smoking or occupational exposure) to facilitate early detection of lung disease [4]. Case-finding strategies based on these recommendations have demonstrated a hit rate of around 20% [5]. However, based on epidemiologic considerations, an estimated 50% of citizens with chronic obstructive pulmonary disease (COPD) in Denmark (approximately 200,000 individuals) remain untreated and most likely undiagnosed in the healthcare system [6]. Whatever the true number may be, underdiagnosis seems to be an issue.

Preventive health checks in the general population is one way to detect individuals at risk. However, low participation rate remains a well-known challenge, especially among persons at risk of developing lung disease [7, 8]. Different approaches to improving early case finding have been investigated [9, 10]. To our knowledge, no previous trials have investigated determinants for improving the participation rate in preventive health checks among persons at risk of developing lung disease, e.g. smokers.

This study aims to investigate if including focused information about the benefits of spirometry in the invitation material may increase the participation rate in a general health check. We hypothesised that the intervention would increase the participation rate by 5% and that more citizens at risk of developing lung disease would choose to participate in the general health check.

## Methods

The study is registered at ClinicalTrials.gov (NCT02615769), and the trial protocol has been published [11]. The study conforms to the Consolidated Standards Of Reporting Trials (CONSORT), including the extension for non-pharmacological interventions and cluster trials [12, 13].

### Design and participants

**Fig. 1:**
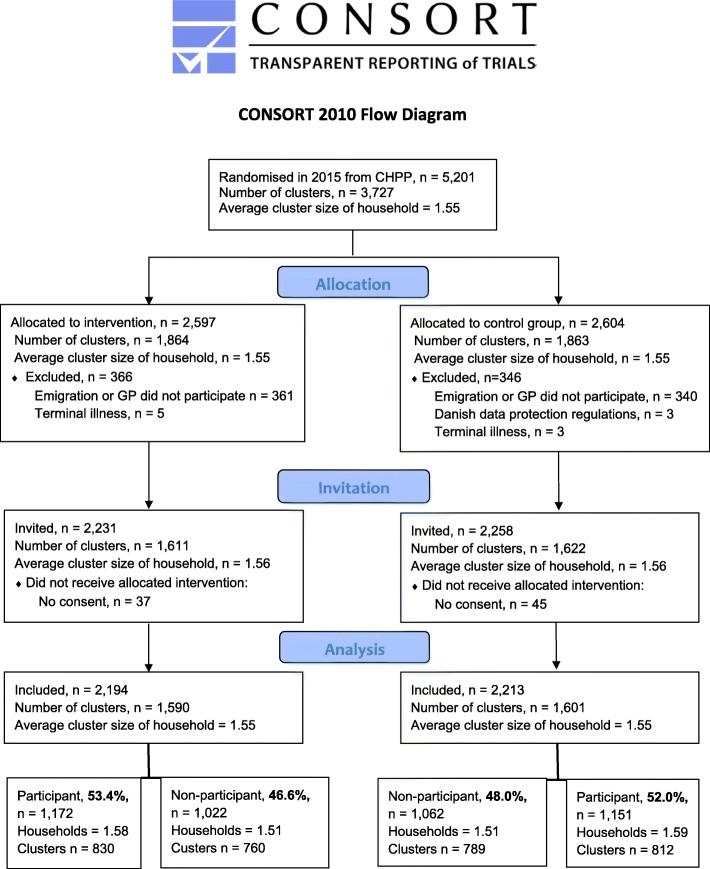
Flowchart of patient inclusion. The study period lasted from 25 November 2015 to 3 February 2017. Abbreviations: GP: General practitioner, CHPP: Check your Health Preventive Programme

We conducted an open-label, household cluster-randomised trial with a two-group parallel design. The trial was embedded in the fourth year of the Danish population-based Check your Health Preventive Programme (CHPP) offering preventive health checks to all citizens aged 30–49 years in Randers Municipality in Denmark [14]. To evenly distribute the workload in the healthcare system, the eligible population (*n* = 26,216) was randomised into five groups of equal size before initiating the CHPP in 2012. The randomisation was conducted on clusters defined by households based on postal addresses from the Danish Civil Registration System [15]. Group four (*n* = 5201) was allocated to the present trial (Fig. 1). A total of 5201 citizens were randomised into either intervention group or control group. The inclusion criteria were: I) living in Randers Municipality within the study period and II) aged 30–49 years. The exclusion criteria were: I) terminal illness reported by the general practitioner (GP) and II) low quality of spirometry measurement. After randomisation, we discovered that 412 eligible citizens had emigrated, and 289 citizens could not be enrolled as their GP operated outside the municipality or did not want to participate in the study. Three citizens were excluded due to risk of identification during the analysis phase. This approach is in line with the statistical guidelines by Statistics Denmark, Danish legislation [16] and the General Data Protection Regulation by the European Commission [17]. Eight citizens were excluded due to terminal illness reported by their GP. This left 4489 eligible citizens for inclusion. Finally, 82 citizens completed the questionnaire and participated in the health check without giving consent for their data to be used in research (Fig. 1).

Invitations were sent out at regular intervals between 25 November 2015 and 13 December 2016. Participants were recruited from 25 November 2015 until 3 February 2017. Invited citizens who had not participated in the clinical examination by 3 February 2017 were listed as non-participants. The study period lasted from 25 November 2015 until 3 February 2017.

### Setting and procedure

The CHPP consisted of two elements: a web-based questionnaire and a clinical examination. A risk profile was generated after completion of both elements. Each participant received a personal recommendation based on the individual risk profile and current health status. The web-based questionnaire consisted of questions regarding smoking, alcohol use, lung symptoms, physical activity, mental health and self-rated health [11]. The clinical examination took place in the community health centre in Randers and included blood analyses, blood pressure, height, weight, cardiorespiratory fitness score and spirometry. Lung function was assessed by EasyOne Diagnostic Spirometer (NDD Medical Technologies, Andover, USA), which was calibrated daily. Spirometer software calculated the FEV_1_/FVC ratio and displayed the predicted value of FEV_1_ and FVC based on reference values. The criterion for correct procedure performance was at least three measurements differing by less than 5%. The spirometry measurements were excluded from the descriptive analysis if this criterion was not achieved (*n* = 36). Abnormal spirometry was defined as FEV_1_/FVC < 0.7. Trained healthcare professionals performed the clinical examinations. The setting, the questionnaire and the clinical examination have been described in detail elsewhere [11, 14].

### Control group

The control group (*n* = 2258) received a standard invitation and an information leaflet by mail. The invitation specified the contents of the general health check and contained practical information, including a link to the webpage, a link to the questionnaire and a prefixed appointment for the health check (date and time). The appointment could be accepted, changed or rejected by phone or internet. If the appointment was not accepted within 7 days, a reminder was sent. Failure to accept within 3 weeks procured another reminder. Invitations and leaflets were dispatched at regular intervals during the study period to evenly distribute the workload in the health centre. Invitations were sent to individuals separately, which means that two individuals in the same household could receive an individual invitation at the same time within the study period. All the material can be accessed in the Additional file 1.

### Intervention group

The intervention group (*n* = 2231) received an altered invitation and a leaflet highlighting the advantages of including a spirometry test in a general health check and provided information about prevention of lung diseases. Furthermore, the citizens were recommended to visit a homepage with additional information and advice on prevention of lung diseases. The material had been developed in cooperation with the Danish Lung Association. Before the invitation was sent, a focus group tested the material for comprehensibility and content, and few revisions were made accordingly. Further details on routine procedures, intervention and information material are available in the Additional file 1 and in the trial protocol [11].

### Sample size

The estimated sample size was 4356 based on the following assumptions: 1:1 randomisation, a two-sided significance level of 0.05, an intra-cluster correlation coefficient of 0.01 [18], a power of 90% and categorical analysis with a power to detect a difference of 5 percentage points in effect size.

### Randomisation, clusters and blinding

An independent data manager performed the randomisation on 7 October 2015 before the inclusion period of the study. The randomisation was based on the postal address, which was obtained from the Danish Civil Registration System [11, 19]. Cluster randomisation by household was chosen to minimise the potential contamination between participants living together because motivation to participate in the health check might affect the entire household. Out of 5201 allocated participants, 3727 households were randomised into either the control group or the intervention group. After randomisation and exclusions, the average cluster size was equally distributed between the control group and the intervention group. Participants were enrolled continuously during the study period if they met the inclusion criteria. The intervention and the outcome were not disclosed to participants. The healthcare professionals also remained blinded. The blinding was unlocked on 3 February 2017 at the end of the study period.

### Outcomes

The primary outcome was participation rate in the general health check measured among the proportion of randomised persons who met the inclusion criteria in each of the two groups. The secondary outcome was the proportion of persons at risk of lung disease in each of the two groups. A person was defined to be at risk if reporting to be a current smoker (daily or part-time) or to have lung symptoms (all of the time or most of the time) in the questionnaire. The clinical outcome data were entered directly into a secure database stored electronically at Aarhus University.

Additional data from Danish national registries [19] were obtained for the population (*n* = 5201), and the unique personal registration number [15] was used to link these data to the clinical measurements. Data on educational level, income level and occupational level were collected from Statistics Denmark [16, 20]. Education level was defined as the highest formal educational attainment categorised according to the United Nation’s Educational, Scientific and Cultural Organization’s International Standard Classification of Education [20, 21]. Education level from 2015 was used to divide the study population into three groups: < 10 (low), 10–15 (medium) and > 15 years of education (high). Income level for 2014 was used and categorised into tertiles and adjusted for family size using the OECD-adjusted income level [22]. Occupational level for 2014 was classified into five groups: employed, self-employed, unemployed, social welfare recipient and others. The Register of Medical Product Statistics, which contains information on all dispensed prescriptions in Denmark since 1994 (ATC codes), was used to investigate if the citizen picked up any respiratory medicine (i.e. short-acting beta agonist (SABA), short-acting muscarinic antagonist (SAMA), long-acting beta agonist (LABA), long-acting muscarinic antagonist (LAMA), or inhaled corticosteroid (ICS)) from a pharmacy in 2012–2015. All data were fully anonymised, and the performed analyses comply with the Danish regulations on register-based research [16].

### Statistical analyses

To compare the baseline characteristics between the intervention group and the control group, descriptive statistics were used. Data are presented as absolute numbers and percentages. The participation rate in the intervention group was compared to that in the control group by estimating the absolute difference in participation rate (PD), and the 95% confidence interval (CI) was calculated using binomial regression with identity link while accounting for household cluster effect by applying cluster robust variance estimation.

Additional analyses were performed using baseline data to compare non-participants in the intervention group with non-participants in the control group. Additionally, the group of excluded persons due to missing data, terminal illness or emigration (*n* = 791) was compared to the group of included participants (*n* = 4489) (Fig. 1). In the additional analyses, we also assessed the impact on participation rate difference if tentatively extending the study period and using 18 months instead of 14 months as cut-off value. Statistical analyses were performed by Stata 14.0 (StataCorp, College Station, TX, USA) [23].

## Results

### Study population

A total of 4489 citizens were invited to the CHPP: 2231 in the intervention group and 2258 in the control group. Of these, 4407 were included in the study: 2194 in the intervention group and 2213 in the control group. The distribution of age, sex, education level, occupation level, income level and respiratory medicine consumption did not differ between the two groups (Table 1). The average cluster size of the household and the number of clusters were equally distributed between the intervention group and the control group (Fig. 1).

**Table 1:** Baseline characteristics of the study population

	Control	Intervention	Total	Missing
	*N* (%)	*N* (%)	*N* (%)	*N*
Total	2258 (50.3)	2231 (49.7)	4489 (100.0)	0/4489
Sex (male)	1142 (50.6)	1143 (51.2)	2285 (50.9)	0/4489
Age groups (years)				
30–34	463 (20.5)	463 (20.8)	926 (20.6)	
35–39	546 (24.2)	572 (25.6)	1118 (24.9)	
40–44	637 (28.2)	605 (27.1)	1242 (27.7)	
45–50	612 (27.1)	591 (26.5)	1203 (26.8)	0/4489
Education (years)				
0–10 (low)	378 (17.1)	386 (17.8)	764 (17.4)	
10–15 (medium)	1143 (51.6)	1104 (50.8)	2247 (51.2)	
15- (high)	692 (31.3)	684 (31.5)	1376 (31.4)	102/4489
Income, 1000 DKK				
Low tertile (0–207)	737 (32.6)	723 (32.4)	1460 (32.5)	
Middle tertile (208–273)	734 (32.5)	740 (33.2)	1474 (32.8)	
High tertile (274-?)	787 (34.9)	768 (34.4)	1555 (34.6)	0/4489
Occupational status				
Employed	1745 (77.3)	1746 (78.3)	3491 (77.8)	
Self-employed	91 (4.0)	86 (3.9)	177 (3.9)	
Unemployed/benefits	68 (3.0)	55 (2.5)	123 (2.7)	
Social welfare recipient	317 (14.0)	310 (13.9)	627 (14.0)	
Others^b^	37 (1.6)	34 (1.5)	71 (1.6)	0/4489
Medicine (Yes)^a^	205 (9.1)	198 (8.9)	403 (9.0)	0/4489

^a^Purchase of respiratory medicine in the last 2 years

^b^ Others include a non-working person from a family relying on one income only

### Primary outcome: participation

A total of 53.4% participated in the intervention group, and 52.0% participated in the control group (difference (diff.) = 1.4% point, 95% CI: − 1.5; 4.4) (Table 2). When stratifying our analyses on different background variables, we found a statistically significant difference in the participation rates for people with a high level of education. For citizens with a high level of education, the participation rate was higher in the intervention group than in the control group (diff. = 5.6% point, 95% CI: 0.4; 10.9). No statistically significant differences in participation rate were found for gender, age group, purchase of respiratory medicine, income or occupation level (Table 2).

**Table 2:** Participation rate, stratified by age group, gender, purchase of respiratory medicine, education level, income and occupational status

	Total	Intervention	Control	Participation
	eligible	participants	participants	rate difference^a^
	(*N*)	*N* (%)	*N* (%)	(95% CI)
Total	4407	1172 (53.4)	1151 (52%)	1.4 (−1.9; 4.7)
Age groups (years)				
30–34	912	221 (48.6)	212 (46.6%)	2.2 (−4.8; 9.2)
35–39	1103	306 (54.4)	273 (50.6%)	3.8 (−2.5;10.1)
40–44	1215	307 (51.8)	322 (51.8%)	0.0 (−6.0; 6.0)
45–50	1177	338 (58.0)	344 (57.9)	0.1 (−5.9; 6.1)
Gender				
Female	2167	590 (55.0)	585 (53.5)	1.5 (−2.7; 5.7)
Male	2240	582 (51.9)	566 (50.6)	1.3 (−2.8; 5.5)
Medicine^b^				
No	4015	1082 (54.1)	1058 (52.5)	1.5 (−1.9; 5.0)
Yes	392	90 (46.6)	93 (46.7)	−0.1 (− 10.1; 9.9)
Education (years)				
0–10 (low)	745	155 (41.3)	139 (37.6)	3.8 (−3.5; 11.0)
10–15 (medium)	2209	574 (52.7)	607 (54.2)	−1.5 (−5.9; 3.0)
16+ (high)	1355	421 (62.5)	387 (56.8)	5.6 (0.0; 11.3)
Income (tertiles)				
Low (0–207)	1429	299 (42.2)	307 (42.6)	−0.5 (−6.1; 5.2)
Middle (208–273)	1446	406 (55.9)	363 (50.4)	5.5 (−0.3; 11.3)
High (274-?)	1532	467 (61.5)	481 (62.2)	−0.7 (−6.3; 4.9)
Occupation				
Employed	3428	971 (56.5)	946 (55.4)	1.1 (−2.6; 4.8)
Self-employed	175	51 (60.0)	50 (55.6)	4.4 (−10.3; 19.1)
Unemployed/benefits	122	21 (38.2)	27 (40.3)	−2.1 (−19.9; 15.6)
Social welfare recipient	615	118 (39.1)	113 (36.1)	3.0 (−4.9; 10.9)
Others^c^	67	11 (33.3)	15 (44.1)	−10.8 (−35.1; 13.5)

^a^Adjusted for cluster size

^b^Purchase of respiratory medicine in the last 2 years

^c^Others include a non-working person from a family relying on one income only

### Secondary outcome: risk profile

A total of 563 citizens (24.2%) participating in the CHPP were at risk of developing lung disease (i.e. current smoker and/or self-reported lung symptoms). We found no differences in the risk profile between participants in the intervention group and participants in the control group (Table 3).

**Table 3:** Characteristics of participants

	Control participants *N* (%)	Intervention participants *N* (%)	Total participants *N* (%)	Missing *N*
Total	1151 (49.5)	1172 (50.5)	2323 (100.0)	0 / 2323
Sex (male)	566 (49.2)	582 (49.7)	1148 (49.4)	0 / 2323
Age groups (years)				
30–34	212 (18.4)	221 (18.9)	433 (18.6)	0 / 2323
35–39	273 (23.7)	306 (26.1)	579 (24.9)	
40–44	322 (28.0)	307 (26.2)	629 (27.1)	
45–50	344 (29.9)	338 (28.8)	682 (29.4)	
Smoking status				
Current smoker	257 (23.0)	264 (23.2)	521 (23.1)	70 / 2323
Never smoke	601 (53.9)	631 (55.4)	1232 (54.7)	
Ex-smoker	257 (23.0)	243 (21.4)	500 (22.2)	
Lung symptoms				
All of the time	9 (0.8)	11 (1.0)	20 (0.9)	38 / 2323
Most of the time	33 (2.9)	27 (2.3)	60 (2.6)	
Now and then	115 (10.2)	115 (9.9)	230 (10.1)	
Rarely	332 (29.4)	330 (28.5)	662 (29.0)	
Not at all	639 (56.6)	674 (58.3)	1313 (57.5)	
Risk profile^a^				
No risk	872 (75.8)	888 (75.8)	1760 (75.8)	0 / 2323
Risk	279 (24.2)	284 (24.2)	563 (24.2)	
Lung function^b^				
FEV_1_/FVC < 0.70	147 (13.2)	147 (13.0)	298 (13.1)	79 / 2323
Income, 1000 DKK				
Low (0–207)	307 (26.7)	299 (25.5)	606 (26.1)	0 / 2323
Middle (208–273)	363 (31.5)	406 (34.6)	769 (33.1)	
High (274-?)	481 (41.8)	467 (39.8)	948 (40.8)	
Education (years)				
0–10 (low)	139 (12.3)	155 (13.5)	294 (12.9)	40 / 2323
11–15 (medium)	607 (53.6)	574 (49.9)	1181 (51.7)	
16+ (high)	387 (34.2)	421 (36.6)	808 (35.4)	
Occupational status				
Employed	946 (82.2)	971 (82.8)	1917 (82.5)	0 / 2323
Self-employed	50 (4.3)	51 (4.4)	101 (4.3)	
Unemployed/benefits	27 (2.3)	21 (1.8)	48 (2.1)	
Social welfare	113 (9.8)	118 (10.1)	231 (9.9)	
Others^c^	15 (1.3)	11 (0.9)	26 (1.1)	
Medicine^d^				
Yes	93 (8.1)	90 (7.7)	183 (7.9)	0 / 2323

^a^Risk of developing lung diseases; current smoker or respiratory symptoms

^b^*FEV*_*1*_ Forced expiratory volume in 1 s, *FVC* Forced vital capacity

^c^Others include a non-working person from a family relying on one income only

^d^Purchase of respiratory medicine in the last 2 years

### Lung function

The highest measurements of FEV_1_ and FVC were used in the analyses as absolute values and as percentage of predicted values; these were based on Danish reference values from a large sample of healthy never smokers [24].

At the clinical examination, we found no difference in lung function between the participants in the two groups (13.0% had FEV_1_/FVC < 0.70% in the intervention group, and 13.2% had FEV_1_/FVC < 0.70% in the control group). The range of FEV_1_/FVC was 37–97%, and the range of FEV_1_ pred. was 34–150%. Due to low quality of spirometry, 15 participants in the intervention group and 21 participants in the control group were excluded. These 36 measurements were also excluded from the sub-analysis (Table 3).

### Additional analyses

No statistically significant differences were identified when we compared non-participants in the intervention group (*n* = 1022) with non-participants in the control group (*n* = 1062). In both groups, we found a higher proportion of social welfare recipients, lower education, lower income and higher consumption of respiratory medicine in non-participants compared to participants (data not shown).

The excluded participants were more likely than were included participants to be social welfare recipients, to have lower education and lower income and to have received more respiratory medicine (data not shown).

Among non-participants, 189 citizens had participated in the study outside the study period. These 189 citizens received the invitation within the study period, but 80 citizens had a clinical examination performed outside the defined study period, and 109 citizens filled out the questionnaire without getting the clinical examination. The 189 citizens were equally divided between the intervention group and the control group. When we tentatively extended the study period, the participation rate increased to 55.6% in the intervention group and to 54.6% in the control group. This rate is similar to the general participation rate in the CHPP [25]. No significant difference was seen in the participation rate between the intervention group and the control group (diff. = 1.0% point 95% CI: − 1.0; 4.0) (data not shown).

## Discussion

### Principal findings

In this cluster-randomised trial, we found that the enhanced invitation material on prevention of lung disease did not influence the participation rate in the general health check. When we stratified the analysis on education, we found a statistically significant difference in the participation rate between the intervention group and the control group for people with long education. This may be a chance finding, but it is also possible that educational attainment might influence the health literacy of the individual citizen and thus the ability to understand the additional invitation material [26]. Furthermore, the proportion of citizens at risk (defined by smoking status and/or self-reported lung symptoms) who participated in the general health check did not differ between the two groups. Additionally, no differences were observed in the number of abnormal spirometry results between participants in the intervention group and participants in the control group.

### Comparison with existing literature

This study is the first to use a cluster-randomised design to investigate the benefits of including targeted information in an invitation to a general health check in order to provide insight into this specific field. Other studies have shown higher participation rates after enhancing invitation material in various settings [27–29]. Our findings are consistent with a study by McDermott et al. [9], who found no effect of enhancing invitations by using question behaviour and financial incentives to increase the participation in a general health check.

We expected a 5 percentage point increase in participation rate based on prior studies [28, 29]. We find this a realistic objective, although we are aware that smokers are known to hesitate to seek help because they feel ashamed and guilty of their self-inflicted diseases [30]. We found no differences in smoking status and lung function between participants in the control group and participants in the intervention group. A proportion of 23% of current smokers was found in each group, which is comparable to the proportion reported in a similar study from the same geographic area and for the same age group [31]. We found 12% to have abnormal lung function in each group, which is higher than expected for a population of this age [6, 32]. In our study, we used the ratio FEV_1_/FVC < 70% to define the level of abnormal lung function. Additionally, we changed the ratio into FEV_1_/FVC < 75% because the cutpoint of a 70% ratio is known to underestimate COPD in young populations and overestimate COPD in the elderly [2, 33]. This change showed no significant difference between the groups: 27.1% in the intervention group and 26.5% in the control group had abnormal lung function (data not shown).

### Strengths and limitations

A major strength of this large-scale study was the randomised design and the analyses following the intention-to-treat principle. Furthermore, our overall attendance rate was 53%, which is high compared to other real-life preventive programmes [34]. This is most likely because appointments were scheduled outside working hours, two reminders were sent, and households received several invitations at the same time to avoid contamination and to increase the total participation rate. Despite the exclusion of 712 eligible citizens after randomisation, we found no significant differences in the baseline characteristics, which contributes to high internal validity. Therefore, the risk of selection bias is considered to be low. Finally, we had the unique opportunity to obtain sociodemographic and medical characteristics about non-participants from the Danish national registers [15, 19], and information bias is thus considered to be low.

Our study also had some limitations. Firstly, there is a risk of misclassification of citizens who present with airway infections like pneumonia or influenza, which could give a false positive result because of impaired lung function. However, we believe this to be of limited significance and to have affected both groups equally. Secondly, we are aware that it is difficult to ensure that the right level of extra information is obtained in the invitation. Too little information in the leaflet about smoking cessation and prevention of lung disease may dilute the intervention, whereas too much information may unnecessarily scare some of the citizens at risk. Thirdly, both groups had similar access to the homepage with information on prevention of lung disease. This may further have diluted the intervention. Therefore, we cannot eliminate that the true intervention effect might have been neglected. Finally, we are aware of the risk of selection bias. A specialist in pulmonary medicine went through the spirometry measurements and excluded 36 measurements due to low quality and technical problems. Due to a low number (*n* = 79) in the overall analyses and an equal distribution among the groups, we believe that this has not affected the results considerably.

### Generalisability of findings

We used a real-life setting, and the participants were a randomised subgroup of the general population in Randers Municipality. Thus, the generalizability of the findings will not only mirror certain citizens at risk, such as current smokers or patients with lung symptoms. The real-life provided high external validity.

In our additional analyses, we found that non-participants in both groups included significantly more social welfare recipients, citizens with lower education, citizens with lower income and citizens receiving more respiratory medicine (data not shown). This is in line with the findings in other CHPP studies, which reported that CHPP participants generally had higher social status than non-participants [14, 25]. Compared to similar municipalities in the same region, Randers has an average health profile for citizens, although higher proportions of obesity, stress-related symptoms and unhealthy diet. Randers also has more current smokers compared to other cities of similar size, but these differences are not statistically significant [31].

## Conclusion

This study revealed that an enhanced invitation was not associated with increased participation in a general health check. Moreover, it was not possible to identify more citizens at risk of developing lung disease. Our study supports previous findings reporting that it is difficult to reach the population at risk of developing lung disease. It might be more beneficial to target the intervention to the population at risk and offer spirometry to specific vulnerable groups of citizens in local communities, e.g. at relevant work places or in socially deprived areas, as also suggested by Larsen et al. [35].

## Additional file

Additional file 1: Invitation, leaflet, and website text to the invitation and comparison group. (PDF 4012 kb)

## Data Availability

The data that support the findings of this study are available from Statistics Denmark and the Danish Health Data Authority, but restrictions apply to the availability of these data, which were used under license for the current study and are not publicly available. However, data are available from the corresponding author upon reasonable request and with the permissions of Statistics Denmark and the Danish Health Data Authority.

## Abbreviations

CHPP: Check Your Health Preventive Program
CONSORT: Consolidated Standards of Reporting Trials
CPR: Civil registration number
Diff.: Difference
FEV_1_: Forced expiratory volume in 1 s
FVC: Forced vital capacity
GP: General practitioner
ICS: Inhaled corticosteroids
LABA: Long-acting beta agonists
LAMA: Long-acting muscarinic antagonists
RCT: Randomised controlled trial
SABA: Short-acting beta agonists
SAMA: Short-acting muscarinic antagonists
